# Endoscopic resection of superficial non‐ampullary duodenal epithelial tumor

**DOI:** 10.1002/deo2.54

**Published:** 2021-09-05

**Authors:** Motohiko Kato, Takanori Kanai, Naohisa Yahagi

**Affiliations:** ^1^ Division of Gastroenterology and Hepatology Department of Internal Medicine Keio University School of Medicine Tokyo Japan; ^2^ Division of Research and Development for Minimally Invasive Treatment Cancer Center Keio University School of Medicine Tokyo Japan

**Keywords:** duodenum, endoscopic resection, outcomes

## Abstract

Although superficial non‐ampullary duodenal epithelial tumor (SNADET) was previously considered a rare disease, in recent years, the opportunities to detect and treat SNADET are increasing. Considering the high morbidity of pancreatoduodenectomy, endoscopic resection can be a treatment option that preserves the organs and contributes maintain patients’ quality of life. Endoscopic mucosal resection (EMR) is a standard treatment for relatively small lesions in gastrointestinal tracts, however, it is difficult because submucosal fibrosis frequently occurs due to the previous biopsy. Recently, some modified EMR techniques including underwater EMR (UEMR) and cold polypectomy (CP) have been proposed. In UEMR, the duodenal lumen is filled with water or saline and resected the targe lesion with a snare without injection into the submucosa. It would be a treatment option that could reduce candidates for ESD especially SNADET less than 20 mm. CP was reported as a safe and convenient means for SNADET. It would also be one of the standard treatments for diminutive lesions, though there remain some concerns on its resectability. ESD for SNADET is technically challenging, especially with an extremely high risk of adverse event (AE) with a reported bleeding rate of more than 20% and perforation rate up to about 40%. However, modified treatment techniques including the water pressure method and pocket creation method have been reported to potentially contribute to improving outcomes of ESD. Moreover, accumulated evidence shows closing the mucosal defect significantly reduces delayed adverse events after duodenal endoscopic treatments. Further studies are warranted to elucidate curative criteria, long‐term outcomes, and appropriate surveillance strategy.

## INTRODUCTION

A superficial non‐ampullary duodenal epithelial tumor (SNADET) was previously considered a rare disease.^1^, ^2^, ^3^, ^4^ Autopsy series have reported its estimated prevalence of 0.02%–0.5%.^5^, ^6^, ^7^ However, in recent years, advances in endoscopic techniques and increased awareness of this disease by endoscopists have led to increased opportunities to detect SNADET.^8^, ^9^

Pancreaticoduodenectomy (Whipple's procedure) is the standard treatment for duodenal cancer. However, the substantial morbidity and mortality rates of Wipple's procedure are 30%–40% and 1%–4%, respectively,^10^, ^11^, ^12^, ^13^ and it is considered too invasive for SNADET.^14^ Endoscopic resection (ER) is an alternative treatment for SNADET, which can preserve the organ and thus maintain the patient's postoperative quality of life. There are two main methods for ER of the duodenum: endoscopic mucosal resection (EMR) and endoscopic submucosal dissection (ESD). Moreover, recently, some modified EMR techniques including underwater EMR (UEMR) and cold polypectomy (CP) have been proposed. In this review, we focus on the current status and recent advances in duodenal ER.

## UNDERLYING ISSUES ON ER FOR SNADET

Since the duodenum is surrounded by various organs such as the pancreas and bile ducts, surgical resections including Wipple's procedure are highly invasive with the substantial morbidity and mortality of pancreaticoduodenectomy (PD) ranges 30%–40% and 1%–4%, respectively.^10^, ^11^, ^12^, ^13^ ER can preserve the organs and contributes maintain patients’ quality of life. On the other hand, ER of the duodenum is technically more difficult and the risk of delayed AE is higher than that of other organs due to various anatomical features as described below. First, the distance of the duodenum from the mouth and the flexure of the lumen (for example, superior and inferior duodenal angles) make it difficult to maneuver the endoscope and sometimes difficult to even approach the lesion. Second, the duodenal wall is extremely thin and can be easily perforated. In addition, a preoperative biopsy may reveal severe fibrosis, making endoscopic resection of even small lesions difficult and sometimes impossible.^15^ Furthermore, due to exposure to bile and pancreatic juice from the duodenal papillae, the risk of delayed AE such as bleeding or perforation is much higher than in other organs, even if the treatment is completed safely.^16^ In fact, previous reports on endoscopic treatment of SNADET have shown that the perforation rate is 13%–50% and the posterior bleeding rate is about 20%,^17^, ^18^, ^19^, ^20^, ^21^, ^22^, ^23^, ^24^ indicating a high risk of accidental injury. In the case of delayed perforation, surgical intervention may require highly invasive surgery such as pancreaticoduodenectomy or prolonged hospitalization even if conservative treatment is possible.

## CONVENTIONAL EMR

EMR is a standard treatment for relatively small lesions in gastrointestinal tracts which consists of mainly 3 steps: local injection into the submucosal layer, strangulation of the target lesion with snare forceps, and resection using electrical current. Even in duodenal EMR, intraoperative perforation is relatively rare, with a reported incidence of around 1%.^22^, ^25^ On the other hand, there was a difference in the incidence of postoperative bleeding, 12% (14 out of 121 cases reported by Nonaka et al.^22^) and 1.4% (2 out of 146 cases reported by Yahagi et al.^25^). Although the detailed reason for the difference in results between these two reports is unclear, one possible factor may be the different lesion sizes. The former study included piecemeal resection of large lesions and the maximum diameter of the included lesions was 50 mm.^22^ Larger mucosal defects after resection may be related to an increased risk of delayed bleeding.

In general, colorectal tumors can be resected en bloc by EMR as far as the lesion size is limited to less than 20 mm in diameter, and ESD is not necessary for these lesions. On the other hand, Yahagi et al. reported that ESD was performed for more than 20% of duodenal lesions less than 20 mm.^25^ One of the reasons why EMR is difficult in relatively small lesions is the fibrosis of the submucosa induced by prior biopsy. In the duodenum, where the mucosa and submucosa are thin, only a tiny biopsy can lead to quite severe submucosal fibrosis, which often results in non‐lifting signs, making snaring impossible even in the small lesion.^15^

## UNDERWATER EMR

**FIGURE 1:**
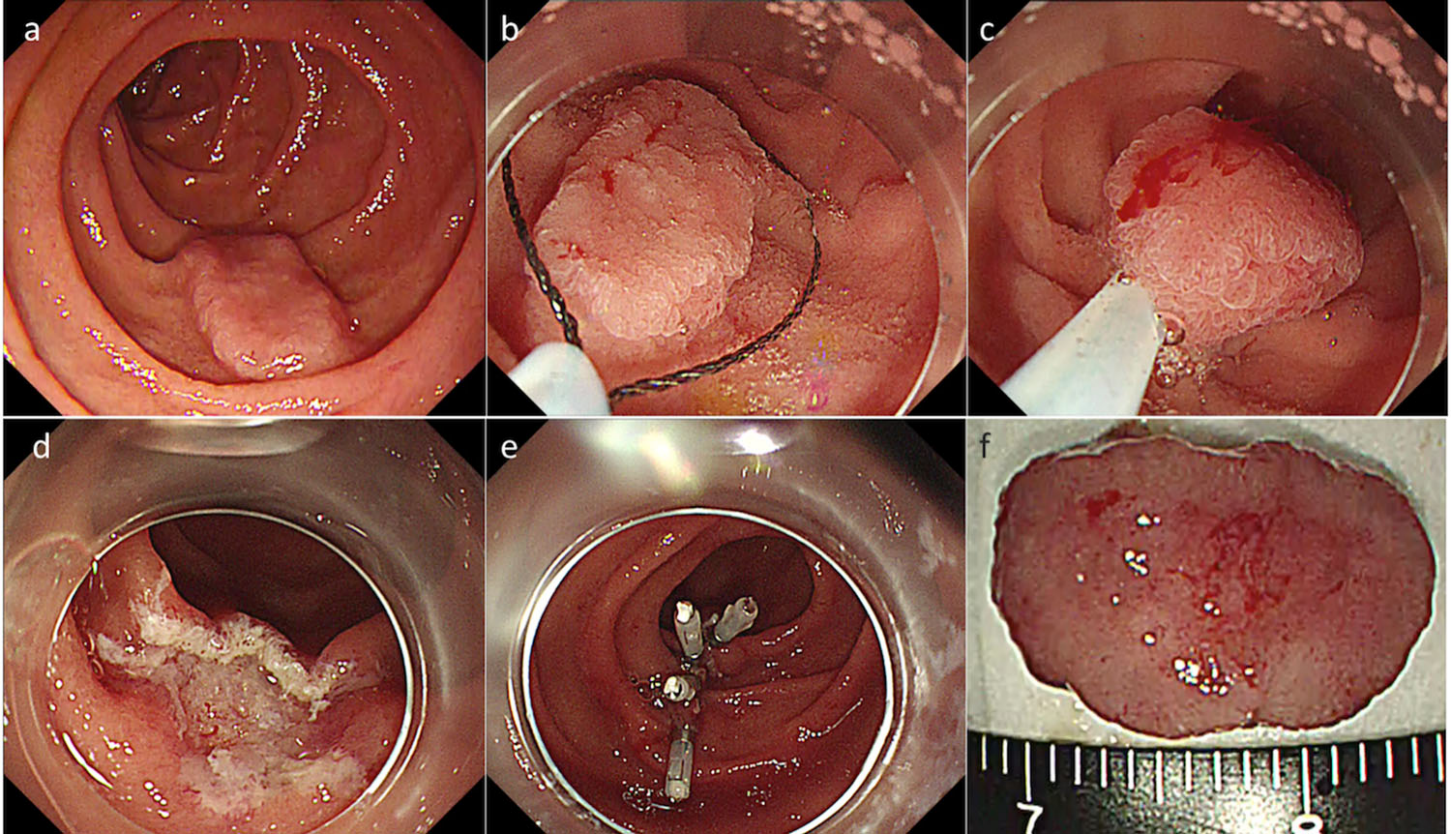
Underwater endoscopic mucosal resection (EMR) for a superficial non‐ampullary duodenal epithelial tumor (SNADET). (a) A 15 mm‐flat elevated lesion is located on descending part of the duodenum. (b, c) The lesion was captured by a snare after filling the lumen with normal saline. (d) The lesion was resected in a single piece without any adverse events. (e) The mucosal defect was completely closed using endoclips. (f) Resected specimen. Pathological findings revealed low‐grade adenoma with free horizontal and vertical margins

UEMR is a new endoscopic procedure in which the duodenal lumen is filled with water or saline and resected the targe lesion with a snare without injection into the submucosa, which was reported by Binmoeller et al.^26^ in 2013 (Figure 1). There is a concern that the risk of perforation might be increased in UEMR by not performing local injection into the submucosa. However, in fact, intraoperative perforation of UEMR is reported to be very rare^26^, ^27^, ^28^, ^29^, ^30^, ^31^, ^32^ (Table 1). This may be because, unlike the semilunar folds of the colon, the duodenal Kerckring folds consist of only mucosa and submucosa and do not contain the muscularis propria. Therefore, the flattened muscularis propria by filling the lumen with fluid, and the difference in specific gravity between the mucosa/submucosa and the muscularis propria reduce the risk of entrapment of the muscularis propria by the snare.

**TABLE 1:** Outcomes of duodenal underwater endoscopic mucosal resection (EMR)

**Author**	**Year**	**Design**	**Number of cases**	**Bleeding**	**Perforation**	**En bloc resection**	**R0 resection**
Binmoeller	2013	Prospective	12	25%	0%	0%	0%
Yamasaki	2018	Prospective	31	0%	0%	87%	61%
Shibukawa	2018	Retrospective	16	0%	0%	88%	NA
Kiguchi	2019	Retrospective	104	2%	0%	87%	67%
Iwagami	2020	Retrospective	162	0.6%	0%	68%	NA
Hirasawa	2021	Retrospective	67	4.5%	0%	93%	76%
Furukawa	2021	Retrospective	28	0%	0%	96%	71%

NA: not applicable

Omitting local injection into the submucosa provides several advantages. First, the ability to obtain a good local injection that facilitates snaring is influenced by the location of the lesion and the skill of the endoscopist, whereas UEMR is not affected by these factors. Second, there are rich blood vessels in the submucosa of the duodenum, and injury by injection needle may cause massive bleeding or hematoma formation in the submucosa. UEMR would avoid this unnecessary adverse event. Third, in the duodenum, the Kerckring fold only consists of mucosa and submucosa and it doesn't contain muscularis propria. Therefore, submucosal injection results in stretching the mucosa and making it difficult to capture the target lesion. Furthermore, one of the most important advantages of this technique is that UEMR reduces the influence of fibrosis in the submucosa by omitting injection. In the duodenum, even a small mucosal biopsy prior to treatment can cause severe submucosal fibrosis.^15^ In fact, Kiguchi et al. reported that 30% of duodenal tumors of 20 mm or less that had been planned to be treated with conventional EMR were actually converted to ESD due to technical difficulty, whereas the conversion to ESD was limited in only 15% in UEMR.^29^ There was also a case of local residual recurrence after duodenal EMR was successfully treated by UEMR.^33^ Moreover, recently a multi‐center prospective study revealed a 97% of the non‐recurrence rate of UEMR.^34^ Based on these findings, UEMR has been regarded as one of the standard therapeutic options for SNADET less than 20 mm.

**FIGURE 2:**
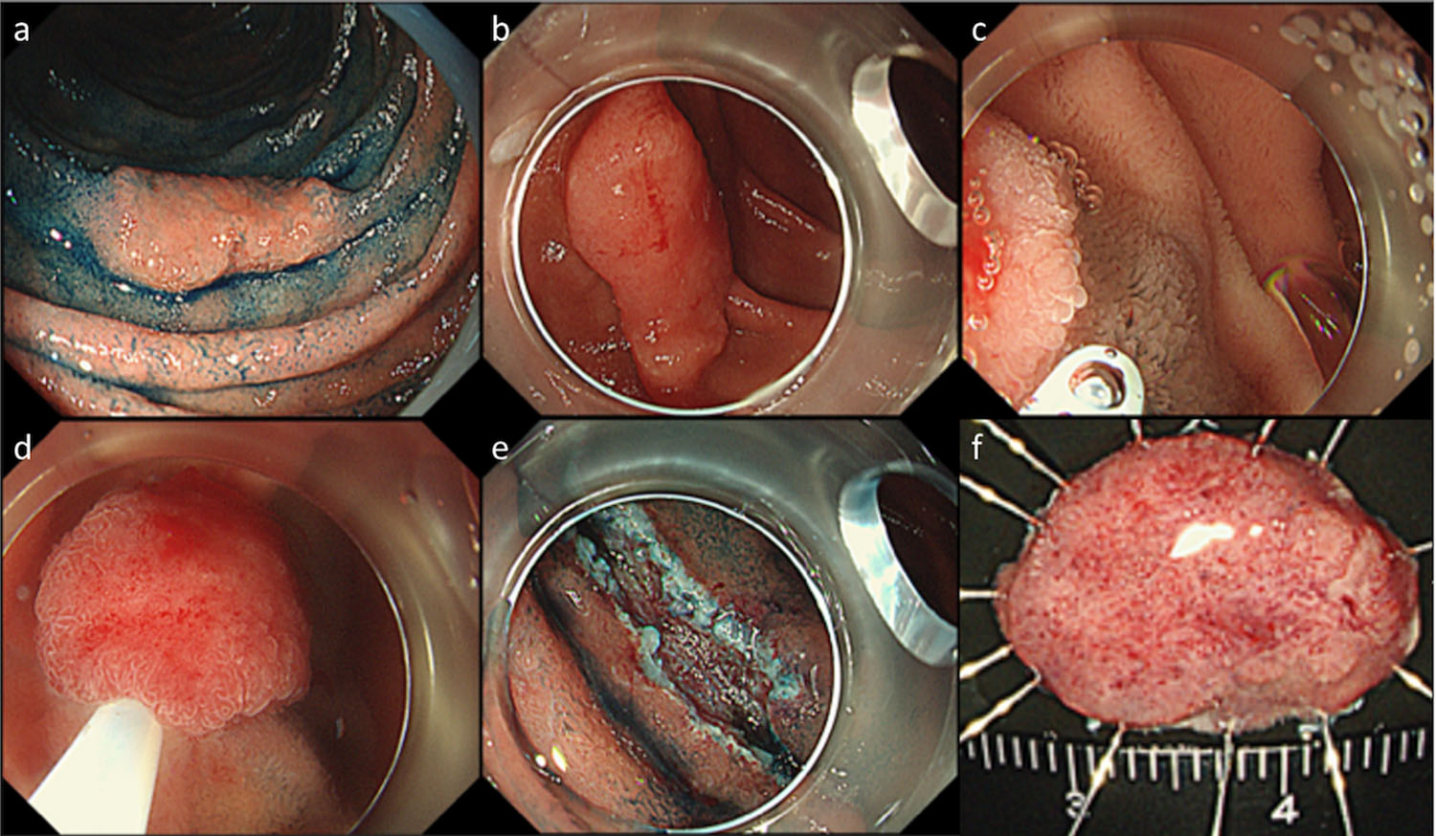
Underwater endoscopic mucosal resection (EMR) with partial submucosal injection for a superficial non‐ampullary duodenal epithelial tumor (SNADET). (a, b) A 15 mm‐flat elevated lesion is located on descending part of the duodenum. (c) Hyaluronic acid was injected only distal edge of the lesion after filling the lumen with normal saline. (d, e, f) The lesion was resected in a single piece without any adverse events. (g) Resected specimen. Pathological findings revealed low‐grade adenoma with free horizontal and vertical margins

On the other hand, one of the issues of UEMR is that the omission of injection may increase the risk of positive lateral margins. Visibility of the anal margin of the lesion during snaring often is impaired due to the duodenal flexure and Kerckring folds and results in residual lesions in that area. Actually, there is a report describing that the proportion of R0 resection is slightly lower than that of conventional EMR, especially for large lesions.^29^ To overcome this, a modified UEMR technique with only partial submucosal injection has been reported^35^, ^36^, ^37^ (Figure 2). The detailed outcomes of this modified technique are still unknown and further studies are warranted.

## COLD POLYPECTOMY

Cold polypectomy (CP) is a recently reported new endoscopic treatment for the gastrointestinal tract which is getting popular along with UEMR. There are two kinds of CP: Cold snare polypectomy (CSP), in which the lesion is physically resected without electric current after strangulation with a snare, and Cold forceps polypectomy, in which the lesion is removed like endoscopic biopsy with jumbo biopsy forceps. It has been suggested that the omission of using electric current in CP may reduce the risk of a late postoperative adverse event, and in fact, it was reported that CSP significantly reduced posterior bleeding compared with conventional EMR with an electric current in patients with colorectal polyps taking anticoagulant medication^38^ and CP has become popular as a safe and convenient treatment method for colorectal adenomas.

**FIGURE 3:**
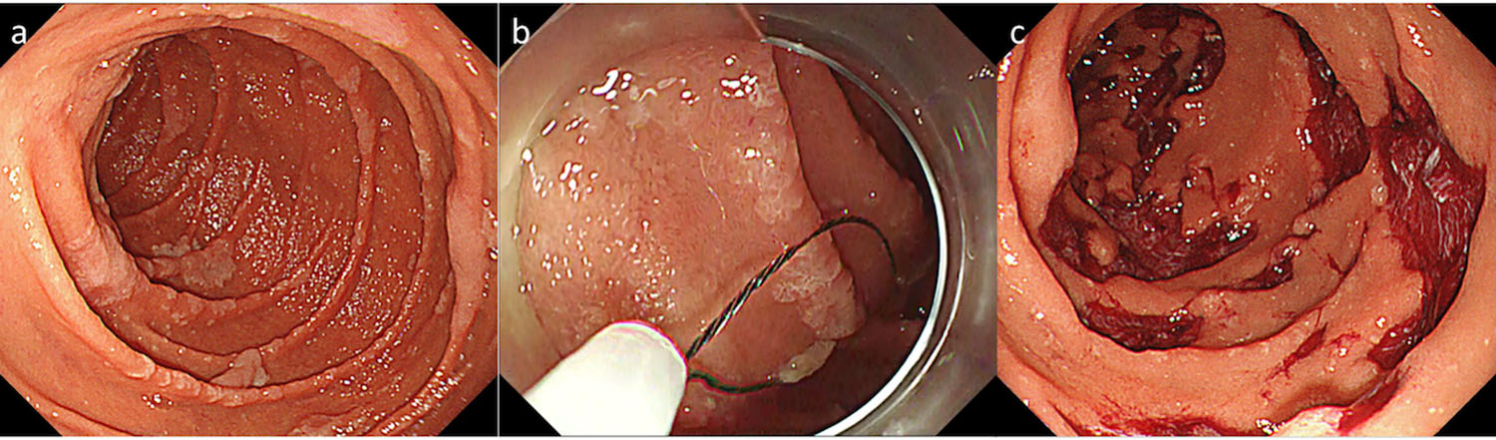
Cold snare polypectomy for multiple adenomatous lesions in patients of familial adenomatous polyposis syndrome. (a) Multiple adenomatous lesions were found in descending duodenum. (b) Lesions were resected by cold snare polypectomy. (c) Post resection mucosal defects. The patient was discharged a day after the procedure without any adverse event

There are also some reports about CP for SNADET. Maruoka et al. prospectively performed CP for 39 lesions in 30 patients and reported there were no AE,^39^ and Hamada et al. performed CSP for 10 patients with familial adenomatous polyposis coli and resected dozens of lesions at once, but reported no serious AE^40^ (Figure 3). Even though these reports are relatively small, and the safety and effectiveness of CP for SNADET should be confirmed further study, it is expected as a promising treatment option, especially for small lesions in the duodenum, where ER is at higher risk of AE than other organs.

On the other hand, there are also some concerns of CP that should be noted. One is an AE unique to the duodenum such as acute pancreatitis. Akimoto et al. reported a case of severe acute pancreatitis that occurred immediately after resection of a small lesion by cold forceps polypectomy locating the oral side of the papilla of Vater. In this case, acute pancreatitis was caused by edema of the minor papilla in a case of pancreatic divisum.^41^ We should pay attention to this rare but serious AE as a precondition for widespread use of CP. The other concern is that CP may be inferior to other kinds of ER in resectability. It has been reported that only the muscularis mucosae and very shallow submucosa are resected in the CSP of the colon.^42^ In fact, there are reports of cases in which local recurrence after CP was found as advanced cancer in the rectum.^43^ Endoscopic diagnosis is less established in the duodenum than in the colon and biological malignancy of SNADET has not been clarified, thus it is still controversial whether to perform CP for all small SNADET uniformly.

## ENDOSCOPIC SUBMUCOSAL DISSECTION

ESD is generally known to provide a secure local cure for early malignancies of the gastrointestinal tract^44^, ^45^, ^46^, ^47^ (Table 2). Similarly, in the duodenum, ESD has been reported to achieve a high en bloc resection rate, regardless of the size of the lesion.^17^, ^19^, ^22^, ^23^, ^24^, ^25^, ^31^, ^48^, ^49^, ^50^, ^51^, ^52^, ^53^, ^54^, ^55^, ^56^, ^57^ Secure local resectability is one of the greatest advantages of ESD for reducing local residual recurrence. In fact, it has been reported that local recurrence occurs up to 30% of large lesions in the duodenum when the resection results in piecemeal resection^22^, ^24^, ^58^, ^59^, ^60^, ^61^, ^62^ (Table 3). Another advantage of en bloc resection is the ability to make an accurate pathological diagnosis, and the high quality of the specimens obtained by ESD makes it possible to pick up subtle findings including slight submucosal invasion or lymphovascular involvement that is difficult to predict by endoscopic observation.

**TABLE 2:** Short‐term outcomes of duodenal endoscopic submucosal dissection (ESD)

**Author**	**Year**	**Design**	**Number of cases**	**Bleeding**	**Perforation**	**En bloc resection**	**R0 resection**
Draganov	2021	Prospective	11	9%	0%	91%	73%
Hirasawa	2021	Retrospective	64	5%	22%	98%	95%
Esaki	2020	Retrospective	55	0	14%	82%	71%
Kuroki	2020	Retrospective	7	14%	14%	86%	71%
Yahagi	2018	Retrospective	174	3%	9%	97%	83%
Tashima	2018	Prospective	50	8%	10%	100%	88%
Matsuda	2017	Retrospective	23	5%	26%	NA	NA
Hoteya	2017	Retrospective	74	15%	28%	99%	88%
Miura	2017	Retrospective	45	9%	16%	98%	82%
Ishii	2015	Retrospective	16	0%	6%	94%	81%
Nonaka	2015	Retrospective	8	0%	25%	75%	50%
Matsumoto	2014	Retrospective	15	20%	20%	87%	87%
Yamamoto	2014	Retrospective	30	0%	9%	100%	90%
Kakushima	2014	Retrospective	13	0%	31%	100%	92%
Jung	2013	Retrospective	14	7%	36%	86%	79%
Endo	2010	Retrospective	5	0%	20%	100%	100%
Honda	2009	Retrospective	9	22%	22%	100%	NA

NA: not applicable

**TABLE 3:** Local recurrence rate of duodenal endoscopic resection (ER)

**Author**	**Year**	**Design**	**Number of cases**	**Treatment**	**Piecemeal resection rate**	**Observation period**	**Recurrence rate**
Hoteya	2017	Retrospective	129	EMR/ESD	10.1%	60.2 months	1.6%
Hara	2019	Retrospective	131	EMR/ESD	10.2%	43 months	3.1%
Nonaka	2015	Retrospective	113	Polypectomy/EMR/ESD	36.0%	51 months	0%
Valli PV	2017	Retrospective	78	EMR	64.1%	33 months	0%
Tomizawa	2018	Retrospective	142	EMR	47.0%	18.8 months	22.5%
Valerii	2018	Retrospective	68	EMR	44.0%	59 months	27.3%
Klein	2017	Retrospective	102	EPMR	100.0%	27 months	17.7%

Abbreviations: EMR, endoscopic mucosal resection; ESD, endoscopic submucosal dissection.

On the other hand, it has been reported that ESD for SNADET is technically challenging, especially with an extremely high risk of AE with a reported bleeding rate of more than 20% and perforation rate up to about 40%^17^, ^19^, ^22^, ^23^, ^24^, ^25^, ^31^, ^48^, ^49^, ^50^, ^51^, ^52^, ^53^, ^54^, ^55^, ^56^, ^57^ (Table 2). Factors that make duodenal ESD technically difficult include the following: maneuverability of the endoscope is limited in the duodenum, which is far from the mouth and fixed to the retroperitoneum. The wall is very thin and the space in the submucosa is narrow due to the rich Brunner's gland. The blood vessels are rich in submucosa, and even a small blood vessel can easily cause major bleeding. Therefore, duodenal procedures should be performed only by highly experienced endoscopists in high‐volume centers. Moreover, factors affecting technical difficulties, such as intraoperative perforation and long time required, have been analyzed, and the presence of lesions at bends such as the superior or inferior duodenal angle (odds ratio, 2.6), lesion diameter exceeding 40 mm (odds ratio, 5.3), and circumference occupied by the tumor exceeding half the circumference (odds ratio, 5.8) were reported as predictors for difficult ESD.^63^

**FIGURE 4:**
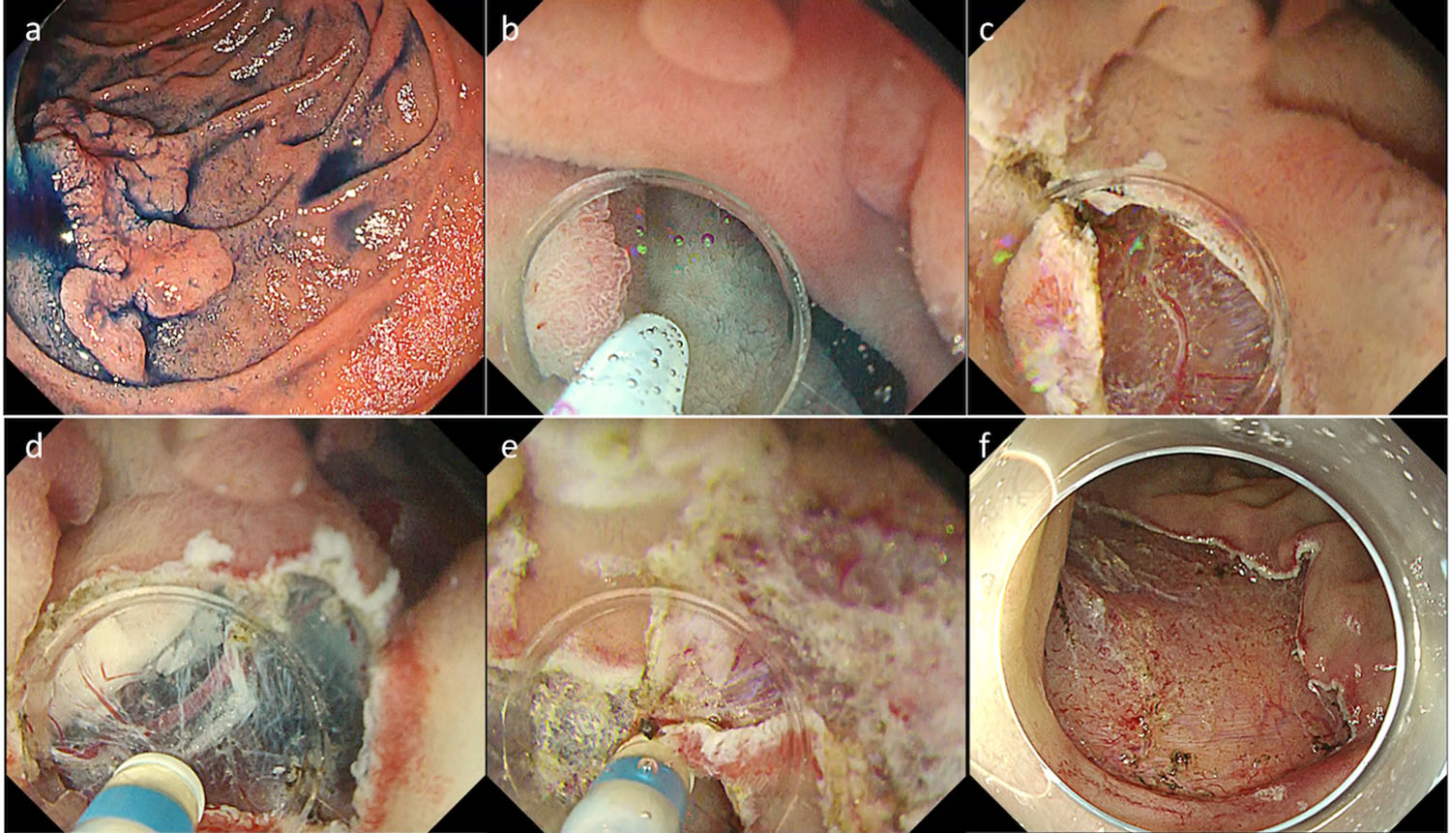
Endoscopic submucosal dissection (ESD) using the water pressure method. (a) A 40 mm‐flat elevated lesion was found in descending duodenum. (b, c) Mucosal incision. (d, e) Hitting the water to submucosa contributed to improved visibility of dissecting area. (f) The lesion was resected without any adverse event

Although duodenal ESD is technically challenging as described above, several improved treatment techniques and newly developed devices have been reported. Yahagi et al. invented a water pressure method in which duodenal lumen is filled with normal saline and an active water stream is utilized to visualize submucosa^64^ (Figure 4). The water pressure method improves the visibility of the submucosa especially in the early stage of submucosal dissection, when it is difficult to directly visualize submucosa, and in the dissection of the lateral edge of the target lesion. The water pressure method was reported to reduce intraoperative perforation and shorten treatment time.^65^ In addition, Miura et al. reported the usefulness of the pocket creation method in duodenal ESD, which utilizing a small tip hood to dissect the submucosa making a pocket without making a full circumferential incision. The pocket creation method is thought to contribute to safety by stabilizing the endoscope and improve the visibility of submucosa.^53^ Dohi et al. also reported the usefulness of the scissors‐type knife for duodenal ESD.^66^ Furthermore, there is also a small case series reporting the usefulness of the traction device.^67^

Although the outcomes of duodenal ESD have improved in recent years due to the above‐mentioned improvement of endoscopic technique and instruments, intraoperative perforation is still considered to be a hazardous AE, sometimes requiring highly invasive intervention including Whipple's procedure. Fukuhara et al. analyzed the clinical course of 32 patients with intraprocedural perforation during duodenal ESD and they found that if the whole mucosal defect including perforation, not only the perforation site, was closed completely, the clinical course was significantly better than those with incomplete closure and was equivalent to those without perforation.^68^

## MANAGEMENT AND PREVENTION OF DELAYED AE

**FIGURE 5:**
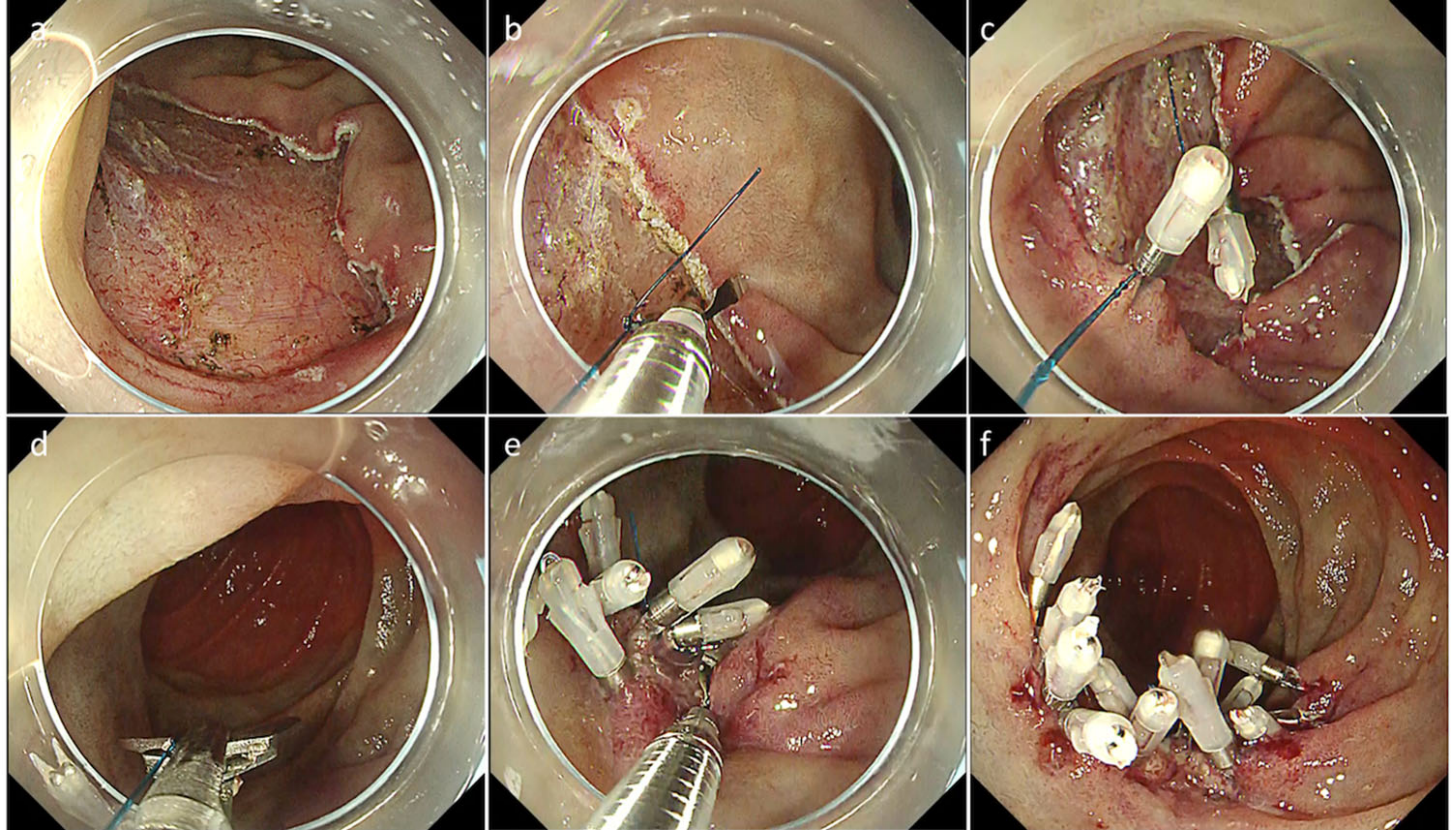
String clip suturing method for a large mucosal defect. (a) A 40 mm‐flat elevated lesion was found in descending duodenum. (b, c) The wound was approximated by pulling a string tightened to the clip. (d, e) The string was cut by scissors forceps and additional clips were deployed. (f) The wound was completely closed

**FIGURE 6:**
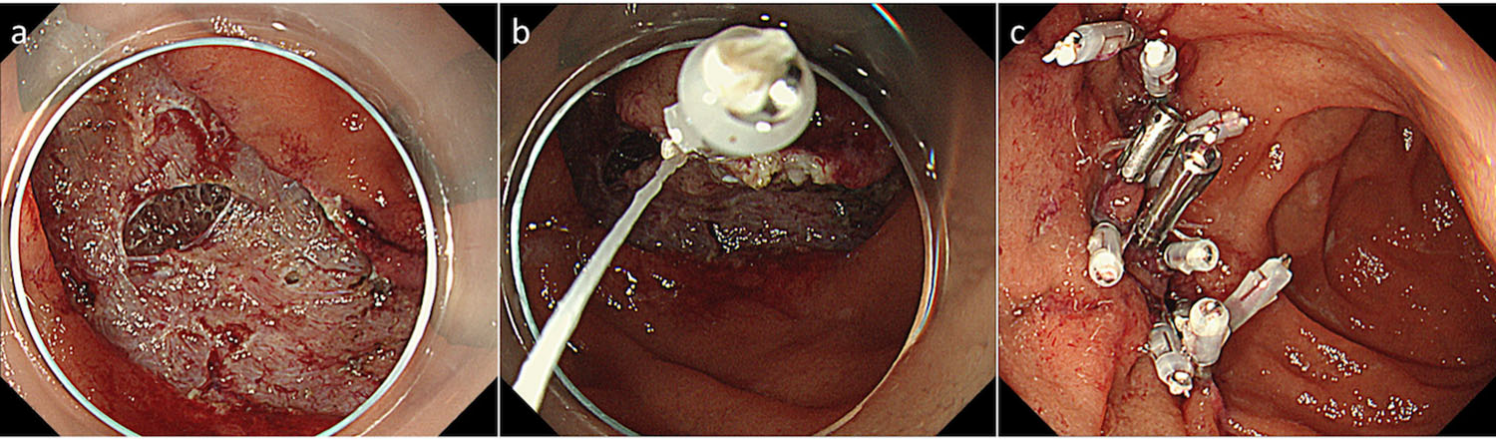
Complete closure of the mucosal defect in the case with intraprocedural perforation. (a) A 5 mm perforation occurred during submucosal dissection. (b) The wound was approximated by pulling string tightened to the clip. (c) The whole mucosal defect was completely closed. The post‐procedural clinical course was non‐eventful and the patient was discharged post procedural day 4

As mentioned above, duodenal ER is at high risk of delayed AE including perforation or bleeding, due to exposure of bile, pancreatic juice, or intestinal juice to the post‐ER ulcer bed.^16^, ^19^, ^22^, ^25^ Various methods have been reported to prevent accidental injury during endoscopic treatment, including simple closure by clips, string clip suturing method (Figure 5), endloop/clips technique, Over‐The‐Scope Clips, or covering with polyglycolic acid sheets.^24^, ^51^, ^69^, ^70^, ^71^, ^72^ Kato et al. reported the proportion of delayed AE was only 1.7% in cases complete closure of the mucosal defect after duodenal ESD was achieved, whereas those in cases with incomplete closure or without closure were 25% and 15.6%, respectively.^69^ Similarly, a recent systematic review analyzing four retrospective studies revealed protection of the post‐ER wound reduced more than 80% of the risk of delayed AE.^73^ Closing the mucosal defect enables to manage the patients conservatively in case of intraprocedural perforation as well as to avoid delayed AE. Fukuhara et al. reported closing the whole mucosal defect, not only perforation site improved clinical outcomes of patients with intraprocedural perforation, and the maximum CRP level and hospital stay were equivalent to those without perforation if the mucosal defect was closed completely^68^ (Figure 6).

Although wound protection after duodenal ER is warranted to prevent delayed incidents, there are also several issues to be solved. Certain kinds of devices and materials are expensive: an Over‐The‐Scope Clips costs 79,800 JPY, the polyglycolic acid sheet itself costs 16,400 JPY, and the fibrin glue costs 34,017 JPY. Moreover, fibrin glue is a blood product derived from donated blood, and there is a low but non‐negligible risk of infection. The string clip suturing method requires only endoclips and a string, those are relatively cheap, however, it is technically challenging and it is sometimes impossible to achieve complete closure even by highly experienced endoscopists, depending on the location and lateral extent of the lesion. Recently, Mizutani et al. analyzed the risk factors for incomplete closure after duodenal ER and they found medial/anterior wall of lesion location and larger lesion size (especially >40mm) were independent predictors for incomplete closure.^74^

Another means to prevent delayed AE is to suture the wound from outside the duodenum by laparoscopy. This novel surgical approach named endoscopic cooperative surgery (D‐LECS) was first reported by Irino et al. in 2015.^75^ Recently a multi‐center retrospective case series including a total of 206 cases underwent D‐LECS revealed favorable outcomes with 95% of R0 resection rate and 4.4% of Clavien‐Dindo classification of three or more (perforation 1.5%, stenosis 1.9%, and bleeding 1%).^76^

**FIGURE 7:**
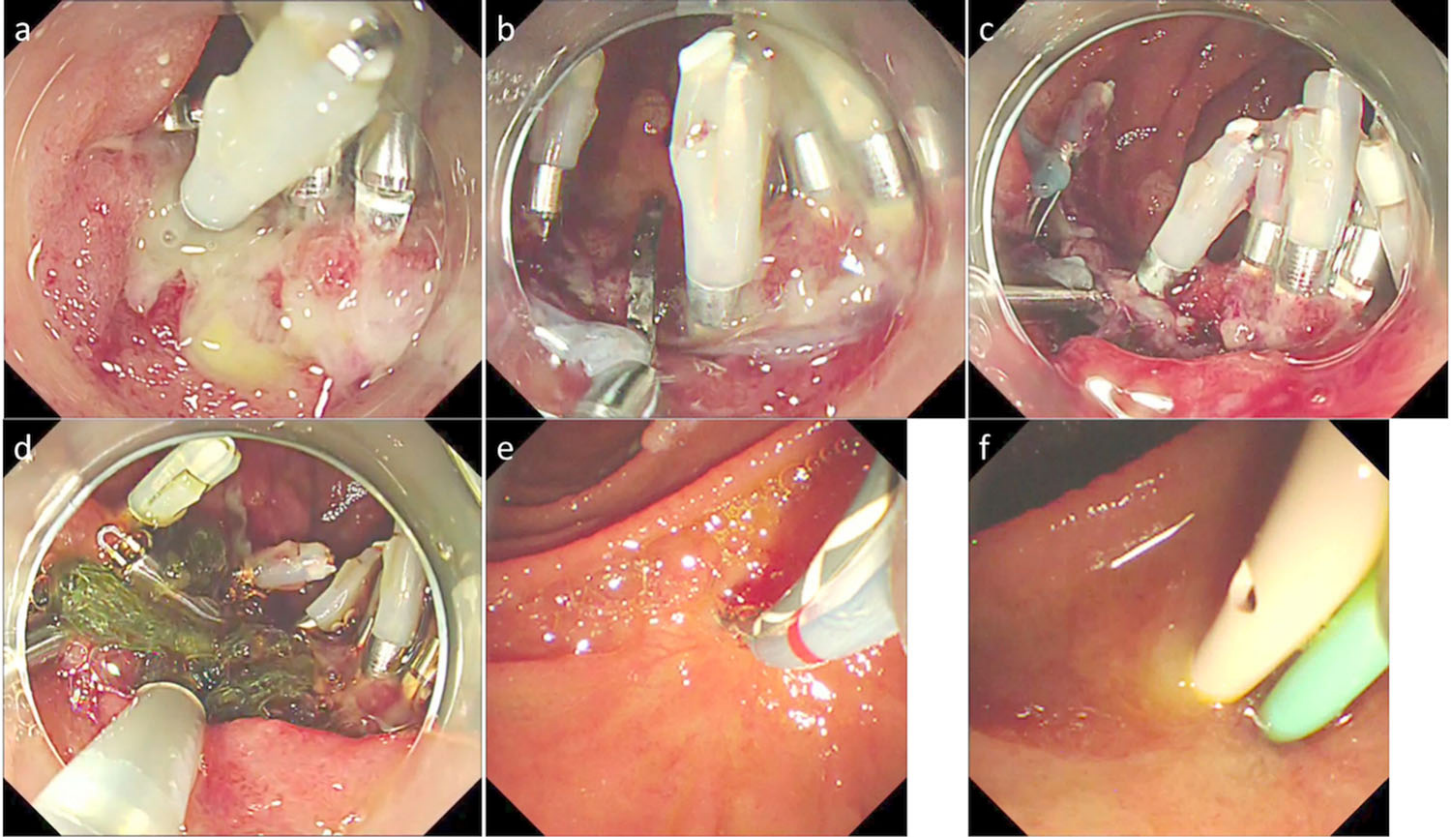
Endoscopic naso‐biliary and naso‐pancreatic drainage (ENBPD) in the case with delayed perforation. (a) Delayed perforation occurred post 2 days after duodenal endoscopic submucosal dissection (ESD). (b, c) We tried to close the perforation with a clip, but due to the fragility of the tissue, the perforation was rather enlarged as a result. (d) Perforated area covered with PGA sheet. (e, f) ENBPD tubes were inserted and the post‐procedural clinical course was non‐eventful without any additional intervention

Moreover, external drainage of bile and pancreatic juice has been suggested for the management of post duodenal ER AE. Fukuhara et al. reported endoscopic naso‐biliary and naso‐pancreatic duct drainage could enable to manage delayed perforation conservatively without any invasive intervention^77^ (Figure 7). Although the incidence of post endoscopic retrograde cholangiopancreatography (ERCP) pancreatitis was 16% in this study, it would be an option for salvage treatment for the cases complete closure of the wound is impossible, considering the high morbidity rate of surgical treatment.

## CRITERIA FOR CURABILITY

Due to the rarity of its incidence, information regarding the curability of ER for SNADET is not still enough. There is no concept of “early duodenal cancer”, on the contrary to other gastrointestinal tract tumors. With regard to the risk for lymph node metastasis (LNM), an analysis of intra‐mucosal duodenal cancer showed no incidence of LNM.^78^ Therefore, intra‐mucosal cancers can be cured ER alone if free resection margin is confirmed pathologically. On the other hand, the incidence of LNM in submucosal cancer is reported to be up to 42%.^78^, ^79^, ^80^ These cases would be candidates for additional surgical resection. However, these studies included only a small number of the lesions with LNM, and the detailed incidence of LNM and its risk factors remains unclear.

## SURVEILLANCE AFTER TREATMENT

Recurrence after endoscopic treatment of superficial duodenal tumors can be divided into local residual recurrence and metachronous multiple lesions. For local recurrence, seven studies including more than 50 cases with a follow‐up period of more than 18 months have been reported. Of these, four were from Japan^22^, ^24^, ^58^, ^60^ and three were from Europe and Oceania.^59^, ^61^, ^62^ The local recurrence rate ranged from 0 to 27.3% and was polarized between low reports (<5%) and high reports (>20%). When these studies were examined in terms of the availability of en bloc resection, all of the reports with high recurrence rates had high piecemeal resection rates.^60^, ^61^, ^62^ Regarding the treatment of locally recurrent lesions, only 0.6% required surgical resection, and most of them could be treated by endoscopic treatment again and there were no reported deaths due to recurrent lesions. The cost of surveillance was reported by Klein et al.,^62^ that endoscopic treatment, including surveillance, was superiorly cheaper than surgical treatment of duodenal tumors.

Regarding the method of endoscopic surveillance, most of the above reports show that the first endoscopy is performed after 6 months–1 year, and if there is no recurrence, follow‐up with endoscopy is performed annually thereafter, suggesting that endoscopic surveillance is necessary at least once a year for patients who underwent segmental resection in the ER. On the other hand, there are currently few reports on heterogeneous multiple lesions, and the details are still unclear.

## FUTURE PERSPECTIVE

For relatively small SNADETs, recently reported new treatment methods, such as UEMR and related techniques and CP, have improved the results. It is expected that these new ER techniques will be widely used as a standard treatment after the long‐term outcomes are elucidated.

Duodenal ESD is still a technically challenging procedure, but one of its advantages is its secure en bloc resection for large lesions. Accumulating evidence suggests wound protection could prevent delayed AE regardless of the presence or absence of intraoperative perforation. It is warranted that a simpler and more reliable method of wound protection will be developed in the future.

We believe that the process of overcoming the difficulty of duodenal ER would contribute to the further progress of endoscopy by discovering unmet medical demands.

## CONFLICT OF INTEREST

The authors declare that they have no conflict of interest.

## FUNDING INFORMATION

None.
